# Deltaretroviruses have circulated since at least the Paleogene and infected a broad range of mammalian species

**DOI:** 10.1186/s12977-019-0495-9

**Published:** 2019-11-27

**Authors:** Tomáš Hron, Daniel Elleder, Robert J. Gifford

**Affiliations:** 1 Institute of Molecular Genetics of the Czech Academy of Sciences, Prague, Czech Republic; 2 MRC-University of Glasgow Centre for Virus Research, 464 Bearsden Rd, Bearsden, Glasgow G61 1QH UK

**Keywords:** Retrovirus, Deltaretrovirus, HTLV, PTLV, BLV, Endogenous retrovirus, Leukemia, Evolution, Paleovirology

## Abstract

The *Deltaretrovirus* genus of retroviruses (family *Retroviridae*) includes the human T cell leukemia viruses and bovine leukemia virus (BLV). Relatively little is known about the biology and evolution of these viruses, because only a few species have been identified and the genomic ‘fossil record’ is relatively sparse. Here, we report the discovery of multiple novel endogenous retroviruses (ERVs) derived from ancestral deltaretroviruses. These sequences—two of which contain complete or near complete internal coding regions—reside in genomes of several distinct mammalian orders, including bats, carnivores, cetaceans, and insectivores. We demonstrate that two of these ERVs contain unambiguous homologs of the *tax* gene, indicating that complex gene regulation has ancient origins within the *Deltaretrovirus* genus. ERVs demonstrate that the host range of the deltaretrovirus genus is much more extensive than suggested by the relatively small number of exogenous deltaretroviruses described so far, and allow the evolutionary timeline of deltaretrovirus-mammal interaction to be more accurately calibrated.

## Main text

The *Deltaretrovirus* genus of retroviruses includes only two extant viral lineages: the primate T-lymphotropic viruses (PTLVs) and an ungulate lineage comprising a single species: bovine leukemia virus (BLV). Both lineages preferentially infect immune cells and exhibit complex regulation of gene expression. PTLVs include several viruses that infect humans, and these human T-lymphotropic viruses (HTLVs) are estimated to infect at least 5-10 million people worldwide [1]. HTLV infection is associated with malignancies and other pathogenic outcomes. Several distinct HTLV species have been described, with each having apparently arisen via a distinct transmission from a non-human primate species [2]. BLV, the only deltaretrovirus known to infect non-primate species, causes enzootic bovine leukosis in cattle [3].

Retroviral infection usually occurs within somatic cells, but occasional infection of germline cells can lead to integrated retroviral genomes being vertically inherited as host alleles called endogenous retroviruses (ERVs). Germline ERV copy number can subsequently increase via a range of mechanisms, giving rise to multicopy ERV ‘lineages’ [4]. Numerous such lineages are present in vertebrate genomes, each being derived from a distinct germline integration event. Some ERV insertions comprise relatively intact proviruses containing internal coding regions flanked by long terminal repeats (LTRs). However, the vast majority are highly degraded and lack viable open reading frames (ORFs). Frequently, LTR-based recombinational deletion occurs, leading to the deletion of internal coding sequences, and leaving behind a single LTR sequence referred to as a ‘solo LTR’ [5].

ERVs comprise a unique source of information about the long-term evolution of exogenous retroviruses [6, 7]. However, the scarcity of ERVs derived from deltaretroviruses prohibits deeper insight into the long-term evolution of this genus. We have previously shown that an ERV sequence in the genome of long-fingered bats (Miniopteridae) derives from a deltaretrovirus that circulated between ~ 45 and 20 million years ago (Mya) [8]. This sequence, labelled ‘Miniopterus ERV a’ (MinERVa) comprises a partially deleted provirus containing a truncated internal coding region flanked by paired LTRs. We subsequently reported sequences disclosing homology to MinERVa LTR region in the genomes of horseshoe bats (Rhinolophidae), indicating that a virus related to MinERVa invaded these species ~ 11–19 Mya [9]. In this study we report the discovery and characterisation of multiple, novel, deltaretrovirus-derived ERVs in mammals.

### Remnants of deltaretroviral ancestors can be found in multiple mammalian orders

**Fig. 1:**
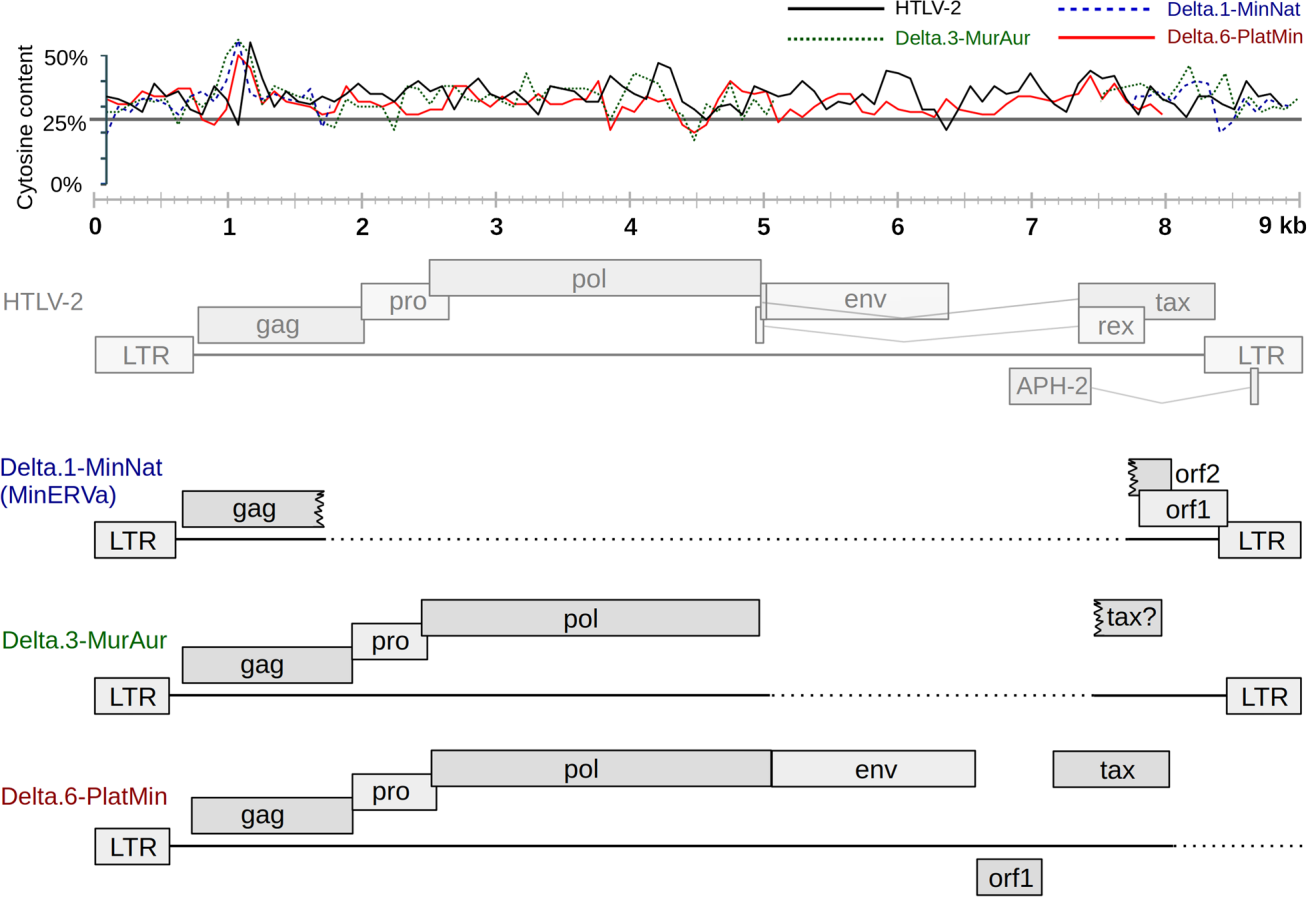
Genomic structure of deltaretrovirus-derived ERVs. ERV genomes are shown schematically using boxes to represent retroviral genes and lines to represent nucleotide sequences. Genes below the lines are in antisense orientation (APH-2 in HTLV-2 and orf1 in Delta.6). Dashed lines represent deleted regions. HTLV-2 genome is used as a reference structure. The plot at the top of the figure shows cytosine content within each genome. This was performed by counting average cytosine content in 100nt windows that overlapped by 10nt

We screened in silico whole genome sequence (WGS) data of 176 mammalian species and unearthed nine novel deltaretrovirus-derived ERVs in multiple distinct mammalian orders, including cetaceans (infraorder Cetacea), carnivores (order Carnivora), insectivores (order Eulipotyphla) and bats (order Chiroptera) (Table 1). To check for possible mistakes in the genomic assemblies we mapped the available short read WGS data to the corresponding contigs. In all cases ERV integrations were covered by mapped reads with no signs of artefactual assembly. Confirmation with polymerase chain reaction (PCR) may ultimately be required to definitely demonstrate that the ERVs we describe are present, and that the sequences of these ERVs are correctly represented in published genome assemblies. However, given the high level of coverage in most of the genomes we examined (see Table 1), and the stringency of current genome assembly algorithms, it is unlikely that the novel sequences we report here represent artefacts. We investigated the genomic characteristics of newly identified ERVs via comparison to the genome sequences of extant deltaretroviruses. Two integrations contain extensive regions of internal coding sequence, while the remainder are solo LTRs. All display the strongly elevated cytosine (C) content typical of deltaretrovirus genomes (average C content > 30% in each solo LTR integration—data not shown; for the two sequences containing internal regions see Fig. 1). The nine ERV sequences detected in our screen together represent six distinct deltaretrovirus lineages, since four solo LTRs identified in distinct carnivore species were found to be orthologous, demonstrating that they originated from a single, ancestral germline integration event.

**Table 1:** Deltaretrovirus-derived ERV loci in animal genomes

Common name	Scientific name	ERV locus ID^a^	Genome Acc.	Scaffold	Coverage	Start	End	Structure
Natal long-fingered bat	*Miniopterus natalensis*	Delta.1-MinNat*	GCA_001595765.1	LDJU01000221	77×	685,870	688,987	Provirus (∆)
Common bent-wing bat	*Miniopterus schreibersii*	Delta.1-MinSch*	GCA_004026525.1	PVJG01030891	24.8×			Provirus (∆)
Rufous horseshoe bat	*Rhinolophus sinicus*	Delta.2-RhiSin*	GCA_001888835.1	LVEH01002092	146.44×	884	224	LTR
Little tube-nosed bat	*Murina aurata*	Delta.3-MurAur	GCA_004026665.1	PVJC01054996	33.3×	2991	9624	Provirus (∆)
Cantor’s roundleaf bat	*Hipposideros galeritus*	Delta.4-HipGal	GCA_004027415.1	PVLB01015338	46.0×	37,888	37,283	LTR
Tailed tailless bat	*Anoura caudifer*	Delta.5-AnoCau	GCA_004027475.1	PVKU01000816	52.5×	300,991	300,731	LTR
Indus River dolphin	*Platanista minor*	Delta.6-PlaMin		RJWK010047772	28×	2710	10,772	Provirus (∆)
Fossa	*Cryptoprocta ferox*	Delta.7-CryFer	GCA_004023885.1	PJEU01009902	46.3×	63,280	63,915	LTR
Common dwarf mongoose	*Helogale parvula*	Delta.7-HelPar	GCA_004023845.1	PJEM01004257	32.3×	95,137	95,687	LTR
Banded mongoose	*Mungos mungo*	Delta.7-MunMun	GCA_004023785.1	PISW01001682	46.7×	85,328	85,862	LTR
Meerkat	*Suricata suricatta*	Delta.7-SurSur	GCA_006229205.1	PITD01006283	50×	94,127	94,678	LTR
Solenodon	*Solenodon paradoxus*	Delta.8-SolPar	GCA_004363575.1	NKTL01022466	26×	630	1220	LTR

^a^Asterisks indicate ERVs that have been described previously

The first near-complete provirus was discovered in the genome of the Indus River dolphin (*Platanista minor*). This sequence is by far the most intact endogenous deltaretrovirus sequence to be reported so far. It comprises a 5′ LTR preceding an internal coding region that contains complete *gag*, *pro*, *pol*, and *env* ORFs, as well as a *tax* gene homolog (Fig. 1, Additional file 1: Figures S1, S2). All of the proviral genome downstream of *tax* appears to have been deleted. In addition, we discovered that, like the PTLVs [10], Platanista ERV encodes an antisense ORF (orf1 in Fig. 1) in the region between *env* and *tax*. Intriguingly, we observed weak, but detectable homology between the putative protein product of this gene and HTLV-2 antisense-encoded gene APH-2 (Additional file 1: Figure S2, [11]).

A second, nearly complete proviral sequence was identified in the genome of the little tube-nosed bat (*Murina aurata*). This ERV comprises paired LTRs and internal coding region spanning *gag*, *pro*, and *pol*, as well as a region encoding a fragment of a putative *tax* accessory gene homolog (Fig. 1, Additional file 1: Figures S2, S3). Previously we proposed the existence of putative accessory gene ORFs in the MinERVa sequence (ORF1/ORF2; Fig. 1), though these genes display no detectable sequence similarity to those of contemporary deltaretroviruses or those detected in Murina and Platanista insertions. The discovery that the Murina ERV encodes a *tax* homolog suggests that the ORF1/ORF2 genes in MinERVa are not divergent versions of *tax* and/or *rex* (as suggested previously), but may instead represent other deltaretrovirus genes.

We also detected four solo LTRs disclosing homology to LTRs of putatively deltaretroviral origin. Two of these were identified in bats, one in an insectivore (Solenodon), while the fourth was found be orthologous in three species of mongoose (family Herpestidae) and the fossa (*Cryptoprocta ferox*), a Malagasy carnivore.

Currently, the genomic contigs harboring the deltaretrovirus-derived ERVs lack host gene annotations. Therefore, we instead attempted to infer the genomic locus via BLAST-based comparisons to annotated mammalian genomes. For the majority of integration sites examined, we did not detect any predicted genes within 10 kb. However, the Anoura ERV is inserted between orthologs of human genes GSKIP and ATG2B, while the Solenodon ERV is downstream of a ZNF10 ortholog.

As far as we have been able to ascertain, all six novel deltaretrovirus lineages reported here, as well as the two that have been described previously [8, 9], are represented by a single copy only. This consistently single copy nature appears to be a unique feature of deltaretrovirus endogenization. We can only draw tentative conclusions here, because almost all published WGS are to some extent incomplete, but based on current information this consistently single-copy nature appears to be a unique feature of deltaretrovirus endogenization. By contrast, endogenous lentiviruses (which also only occur sporadically) typically occur as multicopy lineages in the species that harbour them [12–17]. One possibility is that efficient germline propagation of endogenous deltaretroviruses is prevented by efficient epigenetic silencing in germline cells [18, 19] or by efficient blocking of cell entry receptors by Env proteins encoded by endogenous deltaretroviruses. Alternatively, the toxic effects of deltaretrovirus gene expression may preclude further expansion of germline copy number, such that deltaretrovirus ERVs can only be retained in the germline when they are ‘dead-on-arrival’ (i.e. incapable of expression following integration).

### Phylogenetic analysis of ERV sequences identifies novel deltaretroviral lineages

**Fig. 2:**
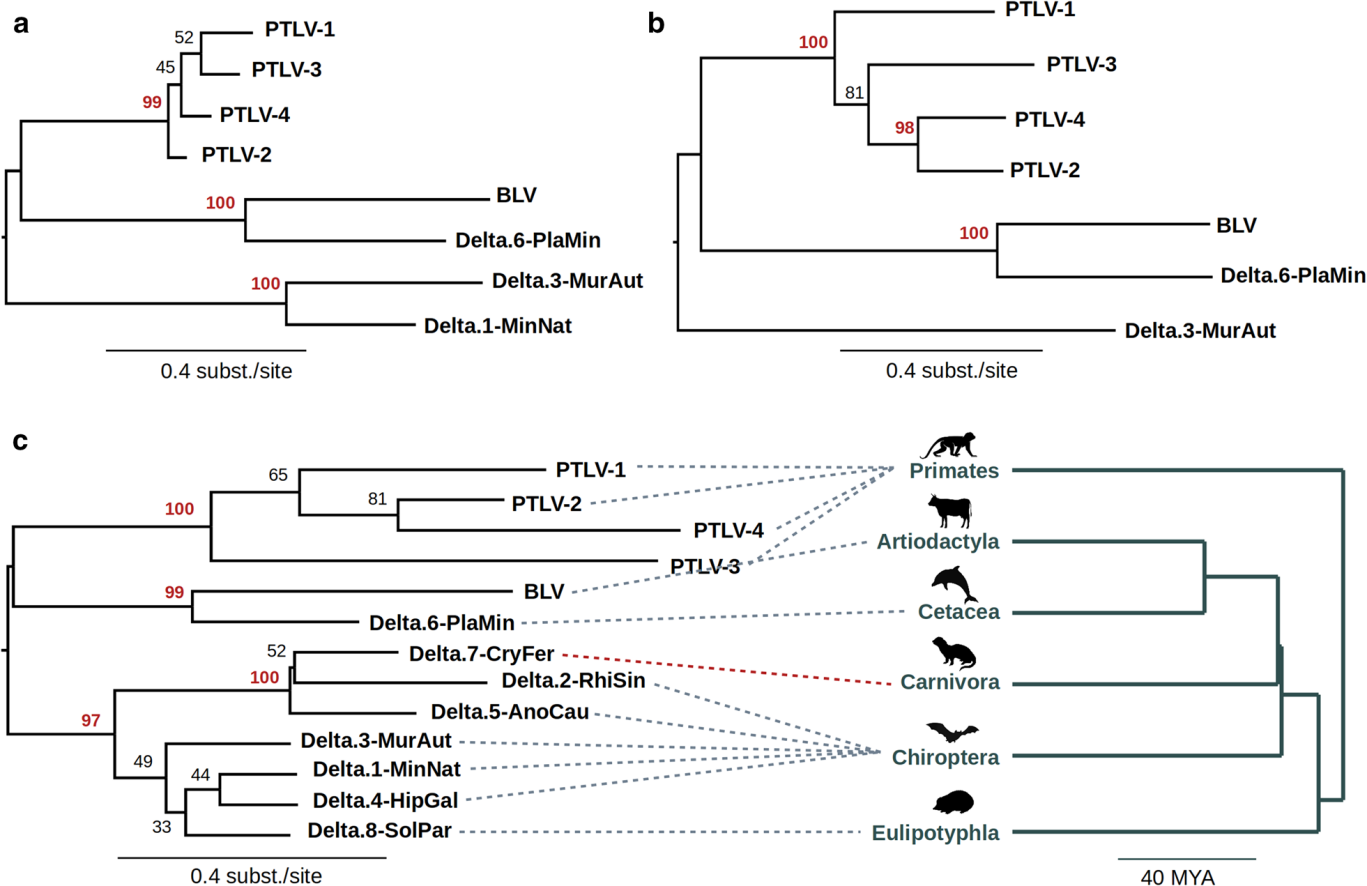
Evolutionary relationships of deltaretroviruses and deltaretrovirus-derived ERVs. Maximum likelihood phylogenies of deltaretroviruses, reconstructed for: **a** the group-specific antigen (gag) amino acid sequence; **b** the polymerase (*p*ol) amino acid sequence; **c** Long terminal repeat (LTR) nucleotide sequences. **c** includes chronogram showing relationships of mammalian orders in which either exogenous or endogenous deltaretroviruses have been reported. Dashed lines connect the hosts with corresponding viruses. Red line indicates potential virus transmission between distinct mammalian orders. Numbers next to nodes show bootstrap support. Support values > 95 are shown in red

For ERVs that spanned internal coding sequences we virtually translated putative ancestral ORFs and aligned the resulting polypeptide sequences with those encoded by exogenous deltaretroviruses. Alignments were used to reconstruct maximum likelihood (ML) phylogenies representing the inferred evolutionary relationships between deltaretroviral *gag* (Fig. 2a), pol (Fig. 2b), and env (data not shown) genes. Since all of these phylogenies exhibited consistent topologies, we found no evidence for ancestral recombination. In the case of the tax gene, sequences were too divergent to support meaningful phylogenetic analysis. The grouping of bat-derived sequences in phylogenies suggests the existence of a bat-specific deltaretrovirus clade, while the grouping of the cetacean sequence with BLV suggests the existence of a deltaretrovirus lineage that infects cetartiodactyls (cetaceans and artiodactyls).

We also reconstructed phylogeny from nucleotide-level alignments of both exogenous and endogenous deltaretrovirus LTR sequences (Fig. 2c). The topology of the resulting tree is consistent with that obtained for the gag and pol genes. Support for branching relationships is weak for some nodes, reflecting the relatively small amount of detectable homology among LTR sequences. Nevertheless, we were able to clearly detect three well-supported clades with bootstrap support > 95%: the primate deltaretroviruses (PTLVs); a lineage of cetartiodactyl viruses comprising BLV and Platanista ERV; and group of bat, carnivora and insectivora deltaretroviruses. Interestingly, this revealed that the solo LTR sequence recovered from carnivore genomes (Delta.7) clusters robustly (bootstrap support 100) within a clade of bat ERVs, suggesting transmission between bat and carnivore hosts has occurred in the past.

Whereas exogenous deltaretroviruses have only been identified in a limited range of species, our results demonstrate the existence of several ancient deltaretroviral lineages, each infecting distinct mammalian groups. The discovery of multiple novel ERV integrations in bat genomes also supports the existence of bat-specific deltaretrovirus clade and suggests that bats possibly represent an important reservoir of deltaretroviruses, or at least did so in the past. This suggests that the host range of modern deltaretroviruses is broader than currently recognised and novel deltaretrovirus species remain be discovered.

### Insights into deep history of deltaretroviruses

**Fig. 3:**
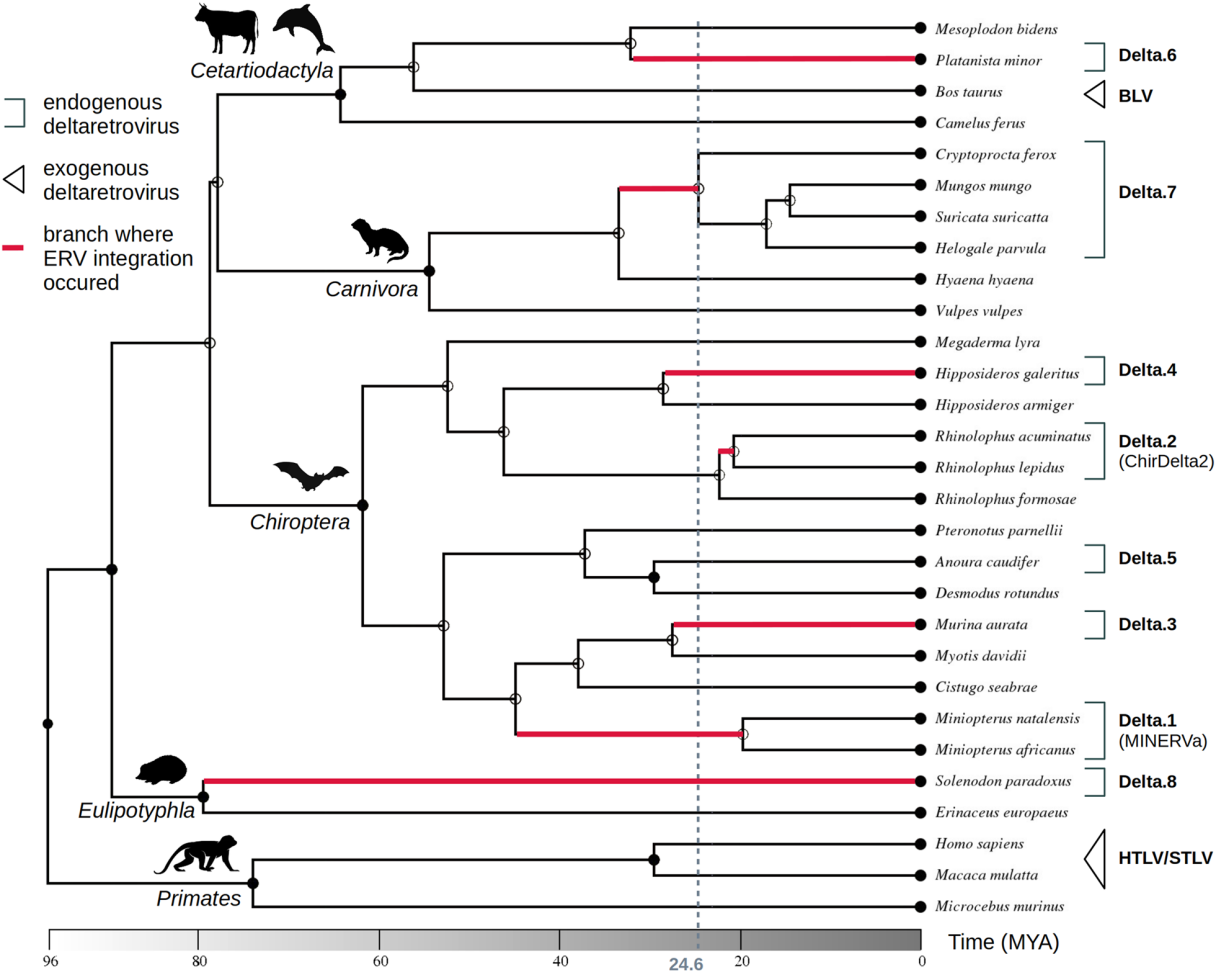
Timeline of deltaretrovirus evolution. A time-calibrated phylogeny of selected mammalian species were obtained from TIMETREE website (http://www.timetree.org/). Occurrence of either endogenous or exogenous deltaretroviruses in the species is indicated next to the species names. Estimated endogenization time intervals are deduced from positivity/negativity of related species for particular ERV integrations. Dashed line indicates the minimal time estimate of deltaretrovirus origin. Closed circles on nodes represent the existence of taxon rank names

The findings of the present study allow further calibration of the deltaretrovirus timeline (see Fig. 3). Firstly, we identify an orthologous solo LTR sequence of apparent deltaretroviral origin in the genomes of mongooses (Herpestidae) and Malagasy carnivores (Eupleridae), demonstrating that this sequence was integrated into carnivore genomes > 24.6 (CI: 20.6–28.7) Mya [20], and therefore pushes the origin of deltaretrovirus genus back to the Paleogene Era or earlier.

The previous studies suggest that deltaretroviral ancestors were infecting bat species sometime ~ 19–45 Mya [8, 9]. The apparent absence of the Hipposideros, Murina and Anoura ERV sequences from other, relatively closely related bat species implies that those integrations originated < 28.4 (20.5–36.2), < 27.5 (23.6–31.6), and < 29.4 (26.9–30.5) Mya, respectively (Fig. 3, [20]). These estimates suggest that bat deltaretroviruses have possibly circulated around the end of Paleogene and beginning of Neogene. In the cases of the Platanista and Solenodon ERVs, genome sequences of closely related species are not available at this time, which results in relatively broad time estimates for germline incorporation (Fig. 3).

We also report unambiguous *tax* gene homologs in the Platanista and Murina ERVs, proving the first evidence that *tax* has ancient origins in deltaretroviruses. In addition, we provide evidence that the Platanista ERV encodes an antisense ORF which discloses some apparent homology to the APH-2 gene of HTLV-2 [11]. This suggests that the use of antisense transcripts is an ancestral feature of deltaretroviruses.

Taken together the data presented here provide the most comprehensive overview of deltaretrovirus evolution to date. Our findings establish that many of the defining features of deltaretroviruses have deep ancestral origins, including cytosine-rich genomes and complex regulation of gene expression via the *tax* gene. Currently eight deltaretroviral ERV lineages have been characterised, in a wide range of mammalian species. It is likely that future availability of thousands more mammalian genomes will reveal additional deltaretroviral ERVs, allowing further insights into deltaretrovirus evolution.

## Methods

### Sequence data and in silico genome screening

The genome sequences of representative deltaretroviruses were obtained from GenBank. Accession numbers as follows: BLV (NC_00141); PTLV1 (J02029); PTLV2 (M10060); PTLV3 (DQ093792); PTLV4 (EF488483); and MinERVa (KY250075). WGS data were obtained from the National Center for Biotechnology Information (NCBI) genomes resource [21]. We obtained all available mammalian genomes as of February 2019. These data were screened for deltaretrovirus-derived ERVs using the basic local alignment search tool (BLAST) program suite, as described previously [12, 22]. Query sequences were derived from exogenous deltaretrovirus genomes and known deltaretrovirus-derived ERVs (MinERVa: KY250075). To identify internal coding regions we used the tBLASTn program with polypeptide sequences as queries. To identify LTRs we used the BLASTn program with LTR nucleic acid sequences as queries. Default parameters were used for all BLAST searches.

### Comparative sequence analysis

The BLAST program [23] and GeneWise tool [3, 24] were used to compare sequences and infer viral ORFs. Translated nucleotide sequences of the deltaretroviral gag and pro-pol regions were aligned using MUSCLE. Alignments were inspected using Se-Al. Low confidence regions were excluded, resulting in an alignment with a total of 244 and 902 positions for gag and pro-pol regions, respectively. Maximum likelihood (ML) phylogeny was generated using PhyML v3.0 [25]. LG model with gamma distribution (four categories) of rates among sites was used as a substitution model. The subtree pruning and regrafting (SPR) operations in an optimized BioNJ starting tree were used for searching of the final tree. Bootstrap support for each node was evaluated with 1000 replicates.

A multiple sequence alignment of deltaretroviral LTR regions was created using the E-INS-i algorithm (suitable for sequences with multiple conserved domains and long gaps) as implemented in MAFFT version 7 [26]. GUIDANCE2 was used to identify and remove unreliable columns in the alignment (Guidance confidence score < 0.2) [27, 28]. The resulting alignment spanned 677 positions. Maximum likelihood (ML) phylogeny was generated using PhyML v3.0 [25]. The K80 model with gamma distribution (four categories) of rates among sites was used as a substitution model. The SPR operations in an optimized BioNJ starting tree were used for searching of the final tree. Bootstrap support for each node was evaluated with 1000 replicates.

## Supplementary information

**Additional file 1.** Additional figures. **Figure S1.** Annotated sequence of Delta.6-PlaMin provirus. **Figure S2.** Local sequence alignments of putative accessory genes of identified ERVs with extant deltaretroviral sequences. **Figure S3.** Annotated sequence of Delta.3-MurAur provirus. **Figure S4.** Annotated sequences of deltaretroviral solitary LTRs identified. **Figure S5.** Global sequence alignment of deltaretroviral LTRs with GUIDANCE alignment confidence score shown.

## Data Availability

All data examined in this study are publicly available via NCBI GenBank

## Abbreviations

PTLV: primate
BLV: bovine leukemia virus
HTLV: human T-lymphotropic virus
ERV: endogenous retrovirus
LTR: long terminal repeat
ORF: open reading frame
MinERVa: miniopterus ERV a
Mya: million years ago
WGS: whole genome sequence
C: cytosine
