# Supplementary material for: Deltaretroviruses have circulated since at least the Paleogene and infected a broad range of mammalian species
Source: Retrovirology. 2019 Nov 27;16:33. doi: 10.1186/s12977-019-0495-9 (PMC6882180; doi:10.1186/s12977-019-0495-9)
Supplement: Supplementary file 1 — Additional file 1. Additional figures. Figure S1. Annotated sequence of Delta.6-PlaMin provirus. Figure S2. Local sequence alignments of putative accessory genes of identified ERVs with extant deltaretroviral sequences. Figure S3. Annotated sequence of Delta.3-MurAur provirus. Figure S4. Annotated sequences of deltaretroviral solitary LTRs identified. Figure S5. Global sequence alignment of deltaretroviral LTRs with GUIDANCE alignment confidence score shown. [file 12977_2019_495_MOESM1_ESM.docx]

**Figure S1**: Annotated sequence of Delta.6-PlaMin provirus

**Figure S2**: Local sequence alignments of putative accessory genes of identified ERVs with extant deltaretroviral sequences.

**Figure S3**: Annotated sequence of Delta.3-MurAur provirus

**Figure S4**: Annotated sequences of deltaretroviral solitary LTRs identified

**Figure S5**: Global sequence alignment of deltaretroviral LTRs with GUIDANCE alignment confidence score shown.
